# Analysis of Asperger Syndrome Using Genetic-Evolutionary Random Support Vector Machine Cluster

**DOI:** 10.3389/fphys.2018.01646

**Published:** 2018-11-21

**Authors:** Xia-an Bi, Jie Chen, Qi Sun, Yingchao Liu, Yang Wang, Xianhao Luo

**Affiliations:** College of Information Science and Engineering, Hunan Normal University, Changsha, China

**Keywords:** genetic-evolutionary random SVM cluster, functional connectivity, classification, Asperger syndrome, abnormal brain regions

## Abstract

Asperger syndrome (AS) is subtype of autism spectrum disorder (ASD). Diagnosis and pathological analysis of AS through resting-state fMRI data is one of the hot topics in brain science. We employed a new model called the genetic-evolutionary random Support Vector Machine cluster (GE-RSVMC) to classify AS and normal people, and search for lesions. The model innovatively integrates the methods of the cluster and genetic evolution to improve the performance of the model. We randomly selected samples and sample features to construct GE-RSVMC, and then used the cluster to classify and extract lesions according to classification results. The model was validated by data of 157 participants (86 AS and 71 health controls) in ABIDE database. The classification accuracy of the model reached to 97.5% and we discovered the brain regions with significant differences, such as the Angular gyrus (ANG.R), Precuneus (PCUN.R), Caudate nucleus (CAU.R), Cuneus (CUN.R) and so on. Our method provides a new perspective for the diagnosis and treatment of AS, and a universal framework for other brain science research as the model has excellent generalization performance.

## Introduction

Asperger syndrome (AS), as a common neurological disease (Gillberg et al., 2016), has an increasing incidence (Jensen et al., 2014) and is usually considered as one of the autism spectrum disorders (ASDs; Lugnegård et al., 2015) or pervasive developmental disorder (Ghaziuddin, 2010; Bucaille et al., 2016). Typically, the patient with AS has a certain degree of cognitive empathy deficiency which is mainly manifested in the weakness of executive function and theory of mind (Montgomery et al., 2013; Oliver et al., 2016). Diagnosing and exploring the pathogenesis of AS is one of the important research fields of brain science (Lorenzo et al., 2016).

In the early exploration of AS, researchers have found that AS and ASD have some similar features including limited interest and repetition, stereotyped activity and communication difficulties (Kamp-Becker et al., 2010; Lugnegård et al., 2013; Woods et al., 2013). But some researches also have noticed the differences between ASD and AS in some aspects such as early speech development (Soulieres et al., 2011), language and intellectual (Radulescu et al., 2013). Therefore, it is necessary to conduct a separate research of AS in subsequent studies. In existing literature on AS, many researchers carried out studies of AS in different perspectives. Rueda et al. (2015) comprehensively used three international common diagnostic criteria for the diagnosis of AS, which effectively improved the stability and accuracy of the diagnostic results. Through the dual approach and voxel analysis, Roine et al. (2015b) found that the white matter (WM) structure in the brain of AS patients was abnormal, and these abnormalities were mainly concentrated in the left ILF region. Tseng et al. (2015) found that there may be structural defects in the amygdala and its related marginal structures. Roine et al. (2015a) discovered that AS had abnormalities in the right caudate and right superior temporal pole base on magnetic resonance images. Channon et al. (2014) observed the social skills of AS patients, and found that they had difficulty in dealing with daily social situations. Abnormal functional mechanisms of emotion detection and emotion differentiation in AS were also found out (Frank et al., 2018). Woodbury-Smith et al. (2005) used the Autism Spectrum Quotient as criterion for discriminating AS and normal person with accuracy of 83%. Neurological soft signs were also applied to discriminate AS and other ASD patients but the performance was unsatisfactory (Hirjak et al., 2014).

It is found that existing researches mainly concentrate on the aspects such as the pathological analysis of the partial AS lesion area (Sato et al., 2014; Di Napoli et al., 2015; Bjørklund et al., 2018), the social adaptability of AS patients (Rodger and Vishram, 2010; Channon et al., 2014; Wilson, 2015), classification of AS and normal people, or AS and other ASD patients (Hirjak et al., 2014; Schwarzová, 2018; Sung et al., 2018). But little literature carries out comprehensive analysis of AS lesions, and makes full use of the unique advantages of the increasingly popular machine learning methods in brain science.

To overcome these shortcomings, we innovatively integrates the methods of cluster and genetic evolution to improve the performance of the model, which called GE-RSVMC. In this study, we acquired functional connectivities between brain regions as sample features, and then randomly extracted samples and sample features to construct the initial SVM cluster. Next, according to the fitness function, the base classifiers in the cluster were selected, crossed and mutated so that the cluster can evolve in the direction of performance improvement. Finally, we used the evolutionary cluster to classify and explore lesions. The cluster is constructed by using multiple base classifiers to enhance the poor performance of a single classifier, and the method of genetic evolution helps to optimize the cluster in efficiency. The accuracy reached to 97.5% and the method effectively ensures the generalization performance of the cluster. The classification accuracy of the model is better than most classification methods, and the lesion brain areas has also been found. As a new trial to explore AS, our research contributes to the diagnosis and treatment of AS.

## Materials and Methods

### Data Acquisition

In this paper, we downloaded the experimental data from the Autism Brain Imaging Data Exchange (ABIDE) database. As an authoritative database of autism research, the data in ABIDE has three modalities which are clinical data, sMRI data and fMRI data. We used 157 participants’ fMRI data to carry out the study including 86 healthy controls (HCs) and 71 AS patients.

The criteria for selecting experimental samples are as follows:

(1) The head motion of the sample is less than 2 mm.
(2) Each experimental participant has the information of full intelligence quotient (FIQ), verbal intelligence quotient (VIQ), and performance intelligence quotient (PIQ) as far as possible.

In order to avoid the interferences of other factors such as age and sex on the results of the experiment, we conducted tests on these factors before conducting experiments. The specific results are shown in Table 1.

**Table 1:** Basic information of AS and HC.

Variables (Mean ± SD)	AS (*n* = 63)	HC (*n* = 72)	*P*-value
Gender (M/F)	54/9	57/15	0.321^a^
Age (years)	13.62 ± 5.50	12.80 ± 2.23	0.246^b^
Full IQ^∗^	96.71 ± 40.16	108.78 ± 12.14	0.017^b^
VIQ^∗∗^	117.16 ± 14.62	108.28 ± 13.15	0.003^b^
PIQ^∗∗∗^	62.16 ± 57.07	107.42 ± 12.95	0.000^b^


*Values are mean ± SD. ^*a*^The *p*-value is obtained by the chi square test. ^*b*^The *p*-value is obtained by a double sample *t*-test. ^∗^ indicates that the scores of eight subjects are missing. ^∗∗^ indicates that the scores of 32 subjects are missing. ^∗∗∗^ indicates that the scores of 28 subjects are missing.*

### Data Preprocessing

Due to the distinctions of participants, we have adopted some preprocessing measures to ensure the unification and standardization of experimental data. DPARSF is used for data preprocessing, and the method used by Cai et al. (2017) provides references for us. The following is the detailed preprocessing process:

(1) Removing the first 10 time points to avoid the environmental inadaptability of the participants in the initial stage of the experiment.
(2) Correcting time slice to ensure that the image data was obtained from the same time.
(3) Adjusting head movements of all subjects to make the brain images of different participants locate in the same position.
(4) Standardizing brain images with the echo-planar imaging (EPI) template to make different participants have identical brain structures.
(5) Smoothing the image by Gauss kernel to reduce noise interference (the full width half height of Gauss kernel was set to 6 mm).
(6) Removing linear drift to decrease the influence of uncertain factors.
(7) Using regression analysis to diminish the impact of covariance such as noise signal.
(8) Filtering uncorrelated blood oxygen level dependent signal with a bandwidth of 0.01–0.08 Hz.

After these procedures, we retained 135 participants including 63 AS and 72 HC, and each participant’s original image was transformed into an image that accords with the research standard.

### Construction of Sample Features

After preprocessing, we continued the follow-up experiment using the preprocessing results of all samples. Firstly, Automatic Anatomical Labeling (AAL) template was used to split the preprocessed brain images into 90 brain regions which are also called as regions of interest (ROI). Secondly, we paired any two brain regions resulting in 4,005 pairs. Thirdly, we treated the Pearson correlation coefficient between the two regions contained in each pair as a functional connection. Finally, each sample would get 4,005 functional connections, which served as the sample features of the following experiment.

### Genetic-Evolutionary Random SVM Cluster

#### The Construction of the Genetic-Evolutionary Random SVM Cluster

Machine learning is increasingly used in brain science. In the existing researches, some scholars used a single classifier to classify AS and HC (Kakihara et al., 2015; Siuly and Zhang, 2016). These methods might usually achieve good classification results for specific types of data, but could not stand out in other situations. To solve the problem, some scholars proposed random classifier clusters, such as the random SVM cluster (RSVMC model) proposed by Bi et al. (2018a). But this method did not fully consider the optimization of the classifier cluster. In order to overcome the drawback, this paper employed GE-RSVMC model. Specifically, we applied the cluster to overcome insufficient generalization performance of a single classifier, and solved the optimization problem of the cluster by using the method of genetic evolution. Then, we could carry out feature extraction and sample classification based on evolutionary cluster. The detailed construction process of the genetic-evolutionary cluster is as follows.

The preparatory step is to divide all the participants into a training set and a test set in accordance with the ratio of 7:3.

The first step is to build a single SVM base classifier. Firstly, we randomly selected a part of samples from the training set as the training samples of a single SVM classifier. Secondly, as each sample has 4005-dimensional features, we randomly selected $\sqrt{4005}$ ≈ 62 sample features as classification features. We used binary coding to represent the selection result of sample features. Specifically, we generate a feature sequence with the length of 4005. If the *i*-th sample feature is selected as a classification feature, the value of *i*-th position in the sequence is set as 1, otherwise the value is set as 0. The classification features sequence (Scf) is given as follows:

(1) $$\mathrm{Scf}={\left\{x_{1},\;x_{2},\;x_{3},\;...x_{n-1},\;x_{n}\right\}}_{n}^{m}$$

where *n* represents the quantity of sample features, and *m* indicates the quantity of sample features be selected. The *x_i_* in Scf is defined by:

(2) $$x_{i}=\{\begin{array}{c} \begin{array}{l} 1\;(the\;i-th\;sample\;feature\;was\;selected) \end{array}\\ 0\;(the\;i-th\;sample\;feature\;was\;not\;selected) \end{array}$$

Finally, we constructed a single SVM base classifier based on the training samples and the classification feature sequence.

The second step is to construct the first generation SVM cluster. We repeated the first step for *p* times to get *p* SVM base classifiers. Then, these classifiers were assembled into a cluster which was called as the first generation SVM cluster.

The third step is to determine the fitness function. In this paper, we used the test set to examine the classification performance of each SVM base classifier, the classification accuracy was considered as fitness function. Thus, the expression of the fitness function is obtained by:

(3) $$F_{j}=\frac{S_{true,\;j}}{S},\;j=1,\;2,\;...,\;L$$

where *S_true,j_* is the quantity of samples which are correctly recognized in the *j*-th SVM base classifiers, and *S* is the totality of samples in the test set. The value of fitness function is a significant index to evaluate the classification ability of the base classifier. The larger the value is, the better the performance of the base classifier will be.

**FIGURE 1:**
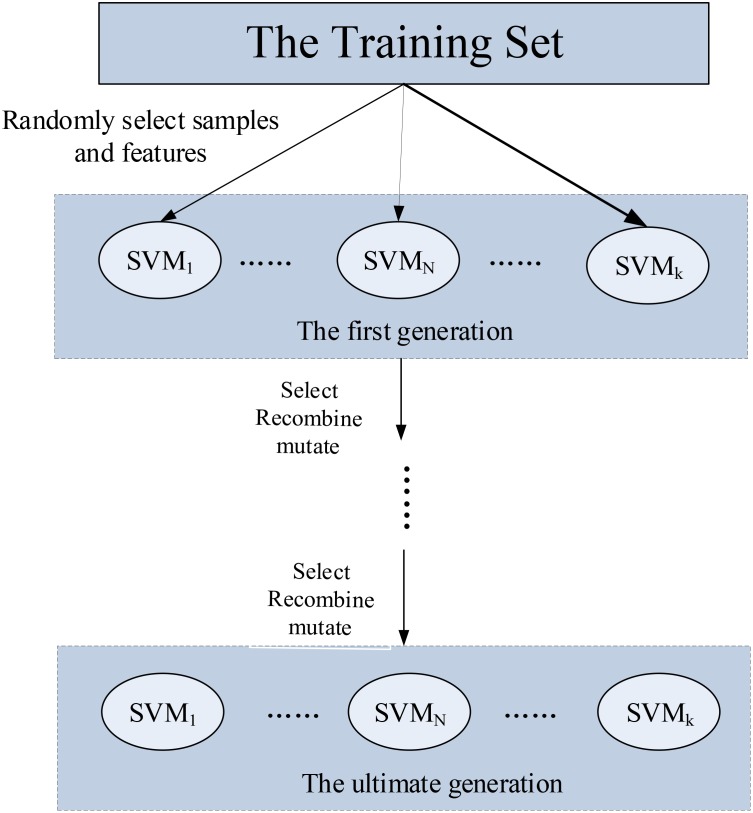
The design of the genetic-evolutionary random SVM cluster.

The fourth step is to implement the genetic evolutionary process of the random SVM cluster. First of all, we selected the base classifier based on fitness function. The base classifier with higher fitness function value is reserved, and the base classifier with lower fitness function value is eliminated. Then, we found out the corresponding feature sequence of each selected base classifier. Finally, we crossed and mutated the feature sequences of these classifiers to generate new offspring feature sequences. The second generation cluster was established based on the new offspring feature sequences. Because we had eliminated the classifiers with lower fitness function, the performance of the second generation cluster was obviously better than the first generation cluster. The process of selection, crossover and mutation is called as genetic evolution. We repeated the process until the classification performance of the cluster became stable. The evolutionary procedure of the cluster is shown in Figure 1.

#### The Optimal Combination of the Times of Genetic Evolution and the Quantity of Base Classifiers

According to the proposed method, we could build a cluster but could not ensure whether it is the optimal cluster. In order to optimize the cluster, we need to find the optimal combination of the quantity of base classifiers and evolutionary times. In this study, the grid search method was used to select the optimal quantity of base classifiers. We used 100–300 base classifiers to build different clusters and searched out the corresponding times of genetic evolution. When both of the quantity of base classifiers and the corresponding evolutionary times are the lowest, the combination is the best.

#### The Classification of the Genetic-Evolutionary Random SVM Cluster

Our first goal is to accurately classify HC and AS through our cluster. As mentioned above, 70% of the experimental samples were used as the training set, and the rest was used as the test set. Each experimental sample has 4,005 sample features and one class label (“+1” or “-1”). “+1” represents HC, “-1” represents AS.

According to the above methods of building the cluster and finding the optimal combination, we could obtain the ultimate cluster. When a test sample enters into the ultimate cluster, each base classifier in the cluster will give a classification result. Because all the base classifiers in the ultimate cluster have excellent classification performance, we assign the equal classification weight to each base classifier. If most of the classification results given by the base classifiers are the “+1,” the samples will be classified as HC, otherwise the sample will be classified as AS.

### Abstracting the Optimal Feature and Brain Region

Our second aim is to explore significantly different features between AS and HC. These differential features are effective in identifying patients and normal people, which we called as “optimal features.” This study used the multi-stage analysis to explore the “optimal features.” The route design for finding the “optimal feature” is as follows.

Step 1: When the optimal quantity of base classifiers was determined and the cluster was evolved to a stable state, we could get the ultimate cluster. Since each base classifier in the ultimate cluster randomly selected 62 sample features, these selected features were the basis to distinguish between AS and HC. We counted the features selected by each base classifier and calculated the frequency of each selected feature. Then, we searched out the most frequent 400 features as “important features.”

Step 2: We extracted the top 70 features from “important features” as “candidate set of optimal features.” Then, we extracted 65 features each time from the “candidate set of optimal features” through stochastic selection to build a RSVMC model, and evaluated the classification performance of RSVMC model by using the test set. Next, we extended the “candidate set of optimal features.” The extraction range of features in “candidate set of optimal features” gradually extends from the top 70 features with the highest frequency to the whole “important feature” set. Finally, we constructed different clusters based on different “candidate set of optimal features,” and the classification accuracies of the clusters were calculated. The “candidate set of optimal features” corresponding to the peak of classification accuracy is the “optimal feature set.”

Step 3: Because each “optimal feature” involves two brain regions, we also used frequency as a criterion to select significant brain regions. We counted each brain region involved in the “optimal features” to get its frequency. The brain regions with higher frequencies are regarded as “significant brain regions.”

## Results

### Construction of Optimal Genetic Evolution Random SVM Cluster

Through many experiments and adjustments, we set the parameters of each SVM base classifier as follows: using the Radial Basis Function (RBF) kernel function, setting cost parameter of RBF as Inf, and setting the gamma parameter as 3.

According to the existing researches, the optimal quantity of base classifier was set up to the interval of [100,300] (Bi et al., 2018b), and genetic evolutionary times was set up to the interval of [0,200] by experiments. Then we used the method of grid search to look for the optimal combination.

**FIGURE 2:**
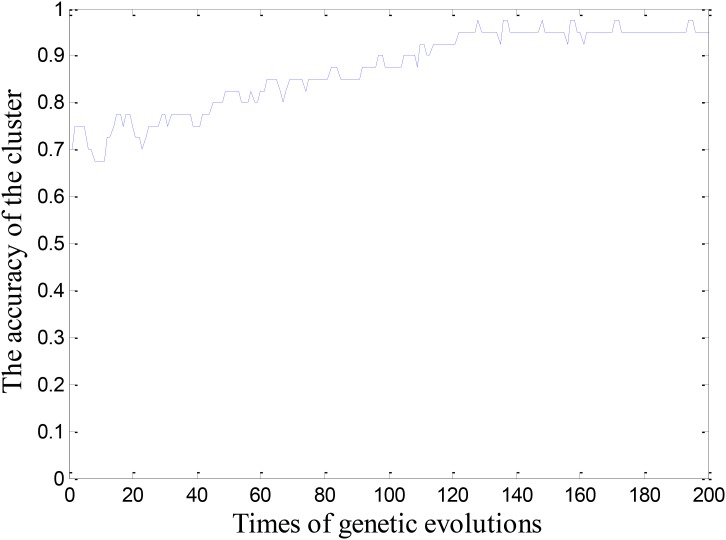
Changes of the accuracy during the genetic evolutionary process.

**FIGURE 3:**
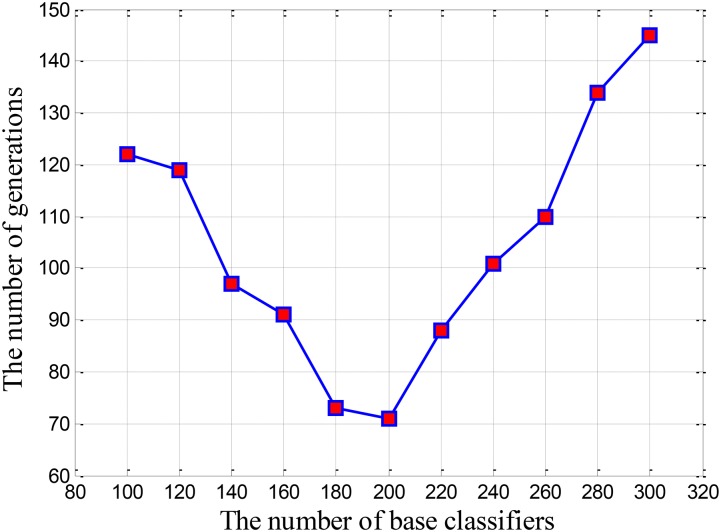
The relationship curve between the evolutionary times and the quantity of base classifiers.

We built a cluster using 100 base classifiers at the beginning of the experiment. In order to make the cluster stable, the cluster was evolved for 200 times, and the evolution process was shown in Figure 2. It is learned that when the cluster is evolved 122 times, the cluster begin to reach a stable state. Thus, we get the first combination of (100,122). Next, we increased the quantity of base classifiers in the cluster with a step of 20, and obtained the corresponding evolutionary times for the cluster. In this way, we can get the combinations under different situations. We regarded these combinations as two-dimensional coordinates and obtained Figure 3. As motioned above, the smaller the quantity of base classifiers is and the less the evolutionary times are, the better the efficiency of the cluster will be. Therefore, we can learn that the coordinate of (200,71) is the optimal combination in Figure 3. Finally, the number of base classifiers in the cluster was set to 200, and the cluster was evolved for 70 times to get the ultimate cluster.

### Classification Performance of Genetic Evolution Random SVM Cluster

**FIGURE 4:**
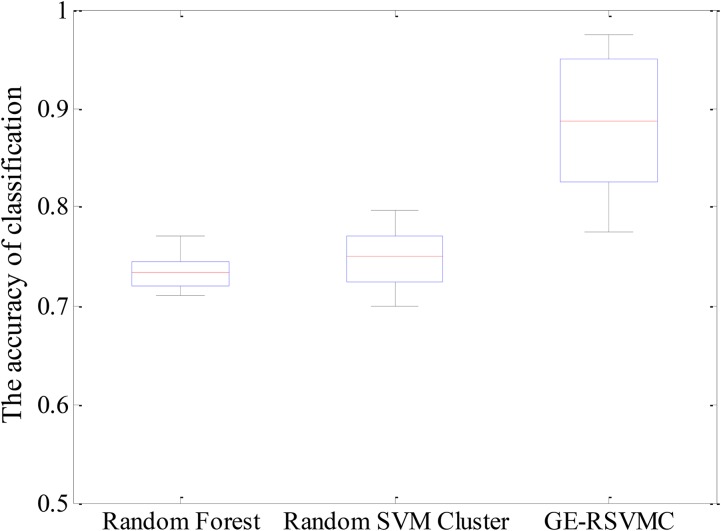
Accuracies of various models.

In this paper, we built the ultimate cluster according to the optimal combination of evolution times and the amount of base classifiers. Our first goal was to accurately discriminate the AS and HC, and we used the test set to evaluate the ultimate cluster. The experimental results showed that the highest accuracy was up to 97.5%. In order to verify the stability of the performance, we conducted experiments for 50 times and the results were compared with those of the Random Forest and the Random SVM Cluster. The detail results are shown in Figure 4 and Table 2. The maximum, median and minimum values of accuracy in our model are 0.975, 0.8875, and 0.775, respectively, which are all superior to the other two methods. On the other hand, the average accuracy of our model is close to 90%, while other methods are less than 75%. We could draw the conclusion from the comparison result that our model has obvious advantages in stability and accuracy.

**Table 2:** Mean accuracies of various model performances.

Model	Accuracy
Random Forest	73.4%
Random SVM cluster	74.6%
Genetic-evolutionary random SVM cluster	88.4%


### Important Features and Optimal Features

**FIGURE 5:**
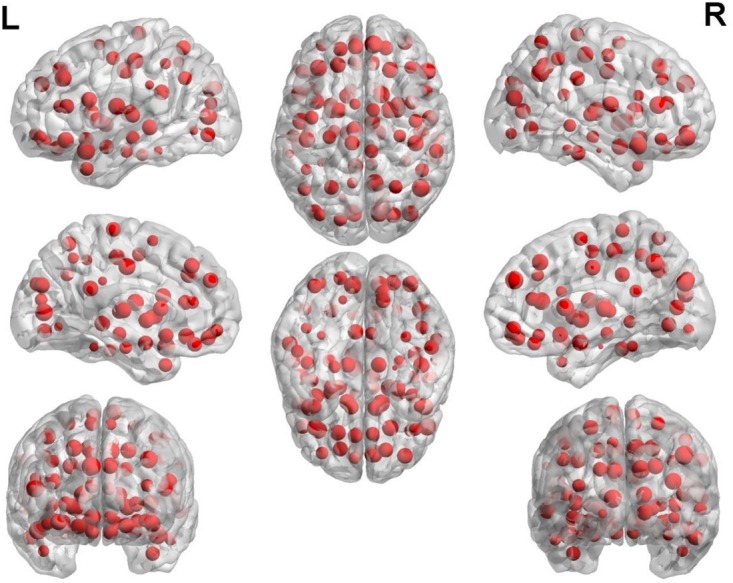
The important features among brain regions.

The second purpose of our research is to find out the differences between AS and HC. These differences have two major functions. One function is helpful for the effective recognition of AS and HC, another is helpful for the pathogenesis analysis of AS from the view of physiological and pathological point. Our study search for these differences by the ultimate cluster. According to the result of grid search, we got the ultimate cluster with 200 base classifiers. Each base classifier randomly selected 62 features as classification features. The occurrence number of each feature in 200 base classifiers was counted. The most frequent 400 features were taken as “important features.” The important features are shown in Figure 5.

**FIGURE 6:**
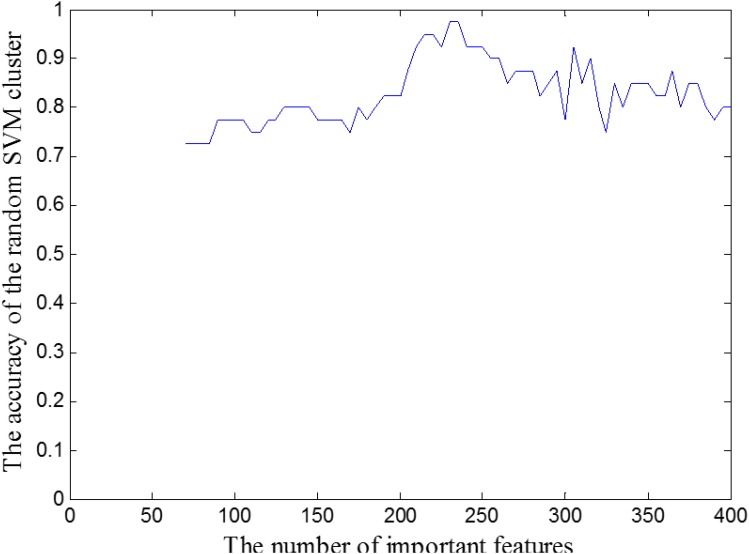
The accuracy of the random SVM cluster with different candidate set of optimal features.

Although “important features” have a good classification performance for HC and AS, they are still not the optimal features for the reason that it may include some unrelated brain areas. Thus we continued to extract the optimal features from “important features.” Specifically, we firstly extract the 70 features from the important features as “candidate set of optimal features.” Next, we randomly extracted 62 features from this candidate set to construct a RSVMC model, and calculated the classification accuracy. Then, we gradually increased the number of feature in “candidate set of optimal features” from 70 to 400 with the step length is 5, and use the same method as above to build different RSVMC. Finally, we calculated the classification accuracies of the RSVMC model under different “candidate set of optimal features” which are shown in the Figure 6. When the number of feature in “candidate set of optimal features” equals to 230, the cluster reaches the peak of accuracy. Thus we regarded the first 230 features of the important feature as the optimal feature set.

### Significant Brain Regions

**FIGURE 7:**
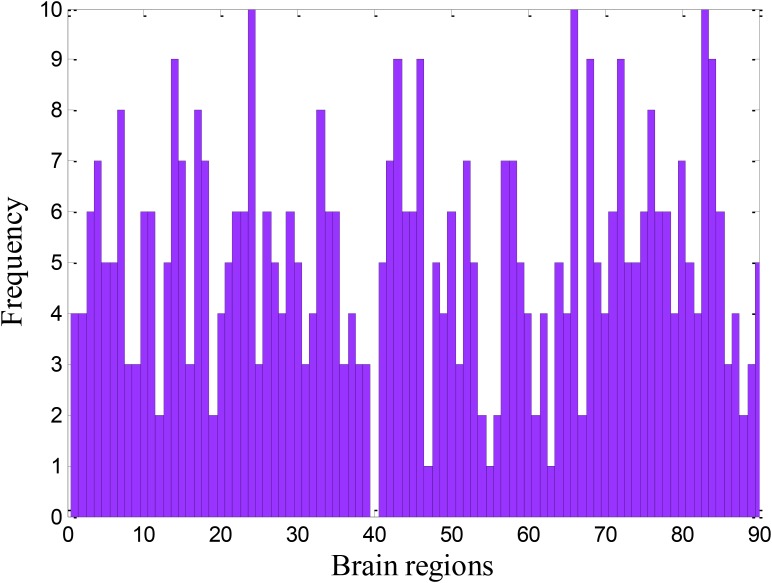
The frequency of the brain region.

Because the sample feature represents the functional connectivity between the two ROIs, the significant ROIs should be detected from the optimal feature set. In this paper, we also used the frequency as a criterion for searching the significant ROIs. We counted the frequency of each ROI in the optimal feature set. The frequency of each ROI is shown in Figure 7. The frequency can detect the correlation between brain regions and disease. The higher the frequency, the stronger the correlation. The ROIs with higher frequencies were used as the significant brain regions. Therefore, we enumerated the information of 13 brain regions with the strongest correlation in Tables 3–5.

**Table 3:** The brain regions with a frequency of 10.

ID of brain region	Brain region	Full name of brain region	Shorter form of brain region
24	Frontal_Sup_Medial_R	Superior frontal gyrus, medial	SFGmed.R
66	Angular_R	Angular gyrus	ANG.R
83	Temporal_Pole_Sup_L	Temporal pole: superior temporal gyrus	TPOsup.L


**Table 4:** The brain regions with a frequency of 9.

ID of brain region	Brain region	Full name of brain region	Shorter form of brain region
14	Frontal_Inf_Tri_R	Inferior frontal gyrus, triangular part	IFGtriang.R
43	Calcarine_L	Calcarine fissure and surrounding cortex	CAL.L
46	Cuneus_R	Cuneus	CUN.R
68	Precuneus_R	Precuneus	PCUN.R
72	Caudate_R	Caudate nucleus	CAU.R
84	Temporal_Pole_Sup_R	Temporal pole: superior temporal gyrus	TPOsup.R


**Table 5:** The brain regions with a frequency of 8.

ID of brain region	Brain region	Full name of brain region	Shorter form of brain region
7	Frontal_Mid_L	Middle frontal gyrus	MFG.L
17	Rolandic_Oper_L	Rolandic operculum	ROL.L
33	Cingulum_Mid_L	Median cingulate and paracingulate gyri	DCG.L
76	Pallidum_R	Lenticular nucleus, pallidum	PAL.R


## Discussion

### Classification Performance

Recently, more and more models of machine learning have been used in the study of neuroimaging. Harlianto et al. (2017) compared several machine learning algorithms for classification, which involved support vector machine (SVM), neural network, decision tree, and naive Bayesian, and the result showed that linear kernel SVM outperformed the others algorithms with an accuracy of 82.35%. Hoang and Nguyen (2018) used SVM, artificial neural network (ANN) and Random Forest (RF) to classify data set, and the accuracy was 87.50, 84.25, and 70%, respectively. Mat Razi et al. (2015) used Multi-Layer Perceptron Neural Network to classify the AS children and typically developing children based on the analysis of EEG signals, and the classification accuracy of the model was up to 79%. Wilson et al. (2014) adopted the SVM to discriminate AS and high-functioning autism (HFA), and the performance of classifier is only 58%.

In this study, the distinction between normal people and patients was abstracted as the binary classification problem in machine learning. We employed a GE-RSVMC for the classification between AS patients and HC. We construct the GE-RSVMC based on ensemble learning, and the ensemble learning model usually has better accuracy and generalization performance than a single classifier. And we introduce the idea of genetic evolution, so that the cluster gradually deletes the classifier with weak classification ability, and retains the classifier with high accuracy. After a certain genetic evolutionary times, each basic classifiers in the cluster has a good classification ability, which makes the cluster achieve a high accuracy. Therefore, the performance of classification stabilizes at the level of 95%, and the highest is 97.5%. Based on the optimal combination of evolution times and the amount of base classifiers, 230 optimal features are retained and used to classify as the optimal feature set. The experimental results show that the GE-RSVMC cannot only distinguish AS patients and HC more accurately, but also identify significant features and the different brain regions between the two groups.

### Analysis of Brain Regions With Greater Frequency

The results of our study indicate that there are some significantly different brain regions including Angular gyrus (ANG.R), Precuneus (PCUN.R), Caudate nucleus (CAU.R), Cuneus (CUN.R), etc. These abnormal regions mean that the lesions occurred in these brain areas. The specific analysis of these brain regions is as follows.

#### (1) Angular Gyrus (ANG.R)

In our paper, the angular gyrus is the one of the ROIs with the highest occurrence number, indicating that the frequency of the abnormal functional connectivity is the highest between this brain region and other brain regions. It plays an essential role not only in the classification of GE-RSVMC model, but also in the pathologic analysis of pathologists.

The angular gyrus locates in the posterior part of the inferior parietal lobule (Seghier, 2013). The angular gyrus involves in semantic processing (Bonnici et al., 2016; Jiang et al., 2018), word reading and comprehension (Pugh et al., 2010), memory retrieval (Price et al., 2015), reasoning (D’Argembeau et al., 2014), and social cognition (Moss and Schunn, 2015). Especially, the increase of the angular gyrus activation makes language ability higher during semantic processing (Ettingerveenstra et al., 2016). And the angular gyrus plays a crucial role in complex information integration and knowledge retrieval (Pick and Lavidor, 2017).

Some studies are in agreement with our results on AS patients. Woods et al. (2013) found that AS patients were commonly characterized by having difficulties in social skills and communication, which can present challenges in everyday functioning. Yanai and Maekawa (2014) found that children with AS had more difficulties with automatic semantic processing than children with normal development. Herrington et al. (2007) indicated that AS patients showed significantly decreased activity between the right superior temporal gyrus and the angular gyrus compared with the HC. Thompson et al. (2010) had observed the decreased activity in angular gyrus and supramarginal gyrus of the right hemisphere in AS with the motor and sensory aprosodias. These results show that the angular gyrus is significantly abnormal in AS, which is of great help to the clinical diagnosis and treatment of AS.

#### (2) Precuneus (PCUN.R)

We discovered that precuneus is one of the vitally important brain regions, and the frequency is ranked second by the occurrence number of 9, which indicating that it makes contribution to the classification of the GE-RSVMC model.

Precuneus is considered as functional pivot of the default-mode network (Utevsky et al., 2014). There is a tight connection between the precuneus and cognitive processes (Fomina et al., 2016), and the precuneus is important in a wide spectrum of highly integrated tasks (Cavanna and Trimble, 2006), including visuo-spatial imagery, episodic memory retrieval and self-processing operations. Then the precuneus plays a role in egocentric spatial processing in the context of memory retrieval (Freton et al., 2014). The results of our study are consistent with some several current researches. For example, Yu et al. (2011) found that the summary AS map indicated lower gray matter volumes in precuneus compared with HC. Radulescu et al. (2013) found a reliance on bottom-up connections in AS among inferior frontal gyri, caudate and precuneus, which contrasted with cognitive control. Sevik et al. (2010) showed that the activation of the precuneus was above the baseline level (*P* < 0.05) for an 18-year-old male with AS and highly unusual calendar memory. These findings demonstrated that the precuneus is abnormal region in AS, which is beneficial to the clinical diagnosis of AS.

#### (3) Caudate Nucleus (CAU.R)

Caudate nucleus is another important brain region in our study, which is one of the prominent brain region detected by the GE-RSVMC model.

The lesions of caudate nucleus lead to impairments in planning and solving problems, mental flexibility, learning, attention, short-term and long-term memory, retrieval, and verbal fluency (Voelbel et al., 2006; Moretti et al., 2017). It is thought to play a role in behavioral monitoring (Schwerdt et al., 2017). Roine et al. (2015a) noted that AS patients have significant degrees of motor incoordination, which sometimes affects writing and drawing skills as well as posture, gait, and gesture incoordination. Some researchers also have the similar results as our findings. Leisman et al. (2014) found that the damage to caudate nucleus was associated with a variety of behavioral abnormalities including organizing behavioral responses and using verbal skills in problem solving. Faridi and Khosrowabadi (2017) noted that the alteration of caudate nuclei in volume was larger by the age in terms of pattern of brain structure in AS than HC. These results show that the study of caudate nucleus is helpful for the treatment of AS.

#### (4) Cuneus (CUN.R)

We also find that Cuneus is a more frequent brain region than remaining brain regions in our study. It is demonstrated that the cuneus plays an important role in classification, and the research of the cuneus is of great significance for the treatment of AS.

The cuneus locates in the occipital lobe (Zhu et al., 2016), and a lot of studies have shown that the cuneus have been related to the visual processing (Vanni et al., 2001; Gershfeld et al., 2015). The visuospatial processing of AS has more supernormal performance in adults than age-matched HC, but which is opposite in children (Chien et al., 2015; Roser et al., 2015). Other researchers such as Herrington et al. (2007) also discovered the difference between AS and HC, which is in line with our results. Cheng et al. (2011), Perkins et al. (2015), and Eilam-Stock et al. (2016) found that the activation of cuneus in AS patients was reduced compared with HC. We can draw a conclusion from the above findings that lesion occurring in the cuneus may lead to AS.

## Limitations

Although our GE-RSVMC model has a great accuracy rate for the classification of AS and HC, there are still some limitations that prevented the model from being more optimized. Firstly, it is simple to use the index of functional connectivity in our study. In a further study, we can also construct other indicators to capture sample features and use these indicators to conduct a more comprehensive analysis of AS, such as multi-classification study of AS and normal people. In addition, considering that we only use the fMRI data of the sample, it is suggested to use the multi-mode data to classify and explore lesions, such as the gene data, structural image data and task-state fMRI. Finally, the performance of the cluster is related to the kernel function of each base classifier. We only use the based classifier with RBF kernel function in the paper. In the subsequent research, we can consider the use of multiple kernel base classifiers to improve the efficiency of the cluster.

## Ethics Statement

Our study was based on secondary analysis of previously collected and publicly available data sets called ABIDE. This study was carried out in accordance with the recommendations of Health Insurance Portability and Accountability Act (HIPAA) guidelines, National Institutes of Health (NIH) Combined Neuroscience Institutional Review Board with written informed consent from all subjects. All subjects gave written informed consent in accordance with the Declaration of Helsinki. The protocol was approved by the National Institutes of Health (NIH) Combined Neuroscience Institutional Review Board. In particular, data of child participants were recruited by Erasmus University Medical Center Rotterdam, ETH Zürich, Georgetown University, Kennedy Krieger Institute, Kennedy Krieger Institute, Trinity Centre for Health Sciences, San Diego State University, Stanford University, and University of California, Davis. Informed assent and consent were obtained from all participants and their caregivers using IRB-approved language and procedures.

## Author Contributions

X-aB proposed the design of the work and revised it critically for important intellectual content. JC, QS, and YL carried out the experiments for the work and drafted part of the work. XL and YW collected and interpreted the data and drafted part of the work. All the authors approved the final version to be published and agreed to be accountable for all aspects of the work in ensuring that questions related to the accuracy or integrity of any part of the work are appropriately investigated and resolved.

## Conflict of Interest Statement

The authors declare that the research was conducted in the absence of any commercial or financial relationships that could be construed as a potential conflict of interest.
